# Transforming Growth Factor Beta 3-Loaded Decellularized Equine Tendon Matrix for Orthopedic Tissue Engineering

**DOI:** 10.3390/ijms20215474

**Published:** 2019-11-03

**Authors:** Susanne Pauline Roth, Walter Brehm, Claudia Groß, Patrick Scheibe, Susanna Schubert, Janina Burk

**Affiliations:** 1 Faculty of Veterinary Medicine, Veterinary Teaching Hospital, Department for Horses, University of Leipzig, D 04103 Leipzig, Germany; brehm@vetmed.uni-leipzig.de; 2 Saxonian Incubator for Clinical Translation, University of Leipzig, D-04103 Leipzig, Germany; claudia.gross@sikt.uni-leipzig.de (C.G.); pscheibe@sikt.uni-leipzig.de (P.S.); susanna.schubert@sikt.uni-leipzig.de (S.S.); Janina.Burk@vetmed.uni-giessen.de (J.B.); 3 Faculty of Veterinary Medicine, Equine Clinic-Surgery, Justus-Liebig-University Giessen, D-35392 Giessen, Germany

**Keywords:** tissue engineering, tendon, scaffold, multipotent mesenchymal stromal cells (MSC), transforming growth factor beta 3 (TGFβ3), surface coating, regeneration, horse

## Abstract

Transforming growth factor beta 3 (TGFβ3) promotes tenogenic differentiation and may enhance tendon regeneration in vivo. This study aimed to apply TGFβ3 absorbed in decellularized equine superficial digital flexor tendon scaffolds, and to investigate the bioactivity of scaffold-associated TGFβ3 in an in vitro model. TGFβ3 could effectively be loaded onto tendon scaffolds so that at least 88% of the applied TGFβ3 were not detected in the rinsing fluid of the TGFβ3-loaded scaffolds. Equine adipose tissue-derived multipotent mesenchymal stromal cells (MSC) were then seeded on scaffolds loaded with 300 ng TGFβ3 to assess its bioactivity. Both scaffold-associated TGFβ3 and TGFβ3 dissolved in the cell culture medium, the latter serving as control group, promoted elongation of cell shapes and scaffold contraction (*p* < 0.05). Furthermore, scaffold-associated and dissolved TGFβ3 affected MSC musculoskeletal gene expression in a similar manner, with an upregulation of tenascin c and downregulation of other matrix molecules, most markedly decorin (*p* < 0.05). These results demonstrate that the bioactivity of scaffold-associated TGFβ3 is preserved, thus TGFβ3 application via absorption in decellularized tendon scaffolds is a feasible approach.

## 1. Introduction

Tendon pathologies, notably injuries of the Achilles tendon and traumatic ruptures of the anterior cruciate ligament, are frequently occurring and long-term musculoskeletal disorders in the current population structure [1,2,3]. High numbers of affected patients, the low-graded level of evidence of conventional treatment options and the insufficient endogenous self-repair mechanisms are driving the need for tissue engineering strategies to restore functionality in injured tendon tissue [4,5].

Orthopedic tissue engineering approaches for functional tissue replacement aim at a detailed reflection of natural conditions. Therefore, the combined application of biocompatible scaffolds with appropriate properties in terms of physicochemistry, mechanical strength and biodegradation with living cells within their corresponding microenvironment has become a generally accepted approach. Additionally, the application of appropriate biomolecular cues in the form of growth factors has proved to be imperative for the interplay between these components and tissue regeneration [6,7]. However, an effective application of growth factors remains challenging due to their short half-life, the susceptibility to inactivation and the potential of rapid dilution [8,9]. Moreover, there might be the risk of side effects following the direct injection of growth factors at the local site of injury. [9]. To maintain a sustained release of growth factors at the site of injury within a reasonable treatment time and under spatiotemporal control, one approach in tendon tissue engineering includes the design of functionalized scaffolds that serve as growth factor delivery systems [10,11].

Growth factors relevant to tendon healing and progenitor cell-mediated tenogenesis include transforming growth factor beta 3 (TGFβ3) [12,13,14]. TGFβ3, which is a member of the TGFβ-superfamily and represents a typical example of a multifunctional and context-sensitively acting growth factor, was noticed to be present during tendon tissue morphogenesis as well as to be actively involved and highly regulated during tendon healing [15,16,17,18,19]. Furthermore, an exogenous application of TGFβ3 in different kinds of cultured cells resulted in the upregulation of tendon-associated genes [16,20,21]. The regulatory duties of growth factors like TGFβ3 in these key processes of tissue development, disease and repair are modulated by interactions with proteoglycans and proteins of the extra- and pericellular matrix [22]. Especially tendon proteoglycans like decorin and biglycan differently bind the three TGFβ-isoforms [22,23]. Thereby, not only activating, but also inhibiting interactions adjust the spatiotemporal availability of growth factors, which is required for the proper regulation of cellular processes.

Carrier systems to deliver growth factors like TGFβ3 include scaffold-free approaches and hydrogels or scaffolds produced from natural and/or synthetic biomaterials [13,24,25]. However, up to now, there have only been occasional reports about the application of decellularized scaffolds used as drug delivery systems in tendon tissue engineering [6,11,13,26,27,28,29].

Due to the preserved tendon-specific extracellular matrix (ECM), decellularized tendon scaffolds excellently mimic the ECM composition and biomechanical properties of native tendon tissue. Since natural tendon tissue is characterized by a particularly high matrix-to-cells ratio, the process of decellularization lead to a natural tendon matrix mainly consisting of hierarchically structured collagen type I. Within this critical fibrillar collagen ultrastructure, inherent bioactive factors of the non-collagenous tendon matrix (glykoproteins and elastin) are preserved. The collagenous fiber-like hierarchical levels of the tendon ECM are aligned along the loading direction of the tissue and confer the characteristically high uniaxial mechanical strength to the tendon ECM. These preserved tissue characteristics modulate cell adhesion, proliferation, and differentiation, as well as biomechanical functionality and biocompatibility of decellularized tendon scaffolds [4]. Taking into consideration that, especially in large tendon defects in humans, the application of autologous, allogeneic or xenogeneic tendon tissue grafts represents a current therapeutic standard, the implantation of functionalized tendon matrices acting as growth factor delivery systems seems reasonable [27,30]. Decellularized tendon scaffolds are established cell carriers to induce the tenogenic differentiation of mesenchymal stromal cells (MSC) in vitro as well as in vivo, and cell-seeded tendon scaffolds have served as tissue grafts in vivo [31,32,33,34]. Functionalized decellularized tendon scaffolds, carrying progenitor cells as well as growth factors, offer the potential to bridge the tendon lesion and to restore functionality rather than to physically replace tendon tissue as ‘placeholder’ with limited regenerative capacities.

The objective of this study was to design a functionally engineered tendon ECM-based construct by loading recombinant human TGFβ3 onto equine decellularized tendon tissue, and to investigate whether TGFβ3 bioactivity is preserved in this context as a first proof of principle. The efficiency of scaffold loading with TGFβ3 via absorption was evaluated by ELISA technique. The bioactivity of scaffold-associated TGFβ3 was compared to TGFβ3 dissolved in the cell culture medium by evaluating its tenogenic effects on scaffold-seeded equine adipose tissue-derived MSC.

## 2. Results

### 2.1. Scaffold Loading with TGFβ3 and Its Efficiency

Prior to any experiments involving tendon scaffolds or cells, possible TGFβ3 trapping by the surface of standard cell culture dishes was evaluated. Indeed, compared to ultra-low attachment dishes, ELISA measurements revealed that 32% (for 5 ng/mL TGFβ3 dilutions) and 59% (for 10 ng/mL TGFβ3 dilutions) had been trapped after 24 h of incubation. Therefore, only ultra-low attachment plates were used for further experiments.

Scaffold loading with either 5 or 10 ng TGFβ3 was successful. ELISA measurements of the rinsing fluids after the incubation of full-thickness or customized 0.3 mm scaffolds with TGFβ3 for 24 h demonstrated that 88 to 94% of TGFβ3 was associated with the scaffold, with no major differences between the scaffold types or TGFβ3 concentrations used. Customized 0.3 mm scaffolds were used in all further experiments on TGFβ3 bioactivity [35]. See Table 1.

### 2.2. Bioactivity of Scaffold-Associated TGFβ3

Indicating its preserved bioactivity, scaffold-associated TGFβ3 had broadly the same effects on equine adipose tissue-derived MSC as dissolved TGFβ3 added as a cell culture medium supplement, which was used as a positive control. This included tenoinductive effects demonstrated by scaffold contraction, cell elongation and an alteration of MSC gene expression, as detailed in the following.

#### 2.2.1. Macroscopic Scaffold Morphology

**Figure 1:**
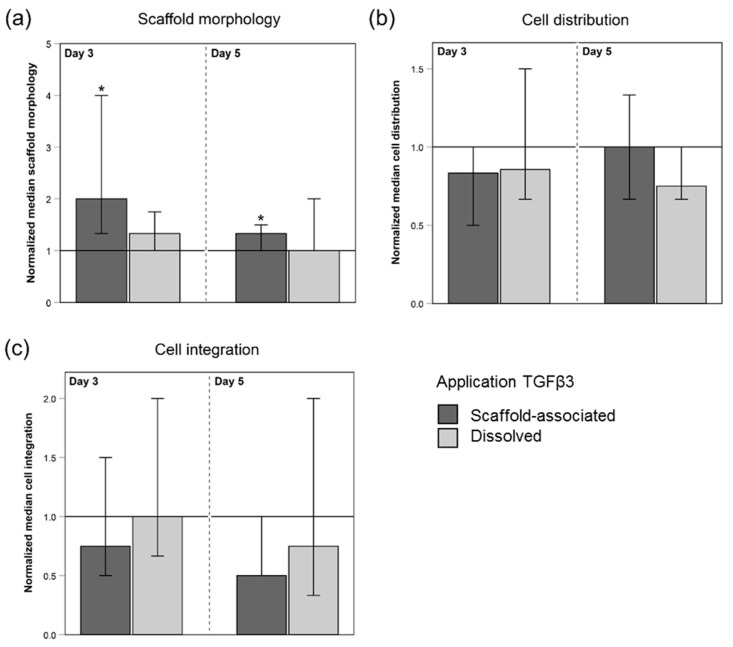
Score points for scaffold morphology (**a**), cell distribution (**b**), and cell integration (**c**) of MSC-seeded tendon scaffolds normalized to the respective internal controls (w/o TGFβ3; indicated by the horizontal line intersecting the *x*-axis at 1.0). MSC-seeded tendon scaffolds were either directly loaded with TGFβ3 (scaffold-associated) or TGFβ3 was added as a cell culture medium supplement (dissolved). The morphology of MSC-seeded scaffolds (**a**) in terms of scaffold contraction was assessed macroscopically. Hematoxylin- and eosin-stained paraffin sections of MSC-seeded scaffolds were microscopically evaluated for cell distribution (**b**) and cell integration (**c**) (10× objective). The scoring systems used are given in the Materials and Methods section. The total number of scaffolds within each group was 42 (*n* = 42). As an exception to this, for the evaluation of cell distribution (**b**) and cell integration (**c**), there were altered numbers of scaffolds due to the technical processing (scaffolds directly loaded with TGFβ3 at day 3: *n* = 41; internal control scaffolds (w/o TGFβ3) in the group of directly loaded scaffolds at day 3: *n* = 39; scaffolds receiving dissolved TGFβ3 at day 5: *n* = 41). Bars indicate the normalized median values and error bars the 95% confidence interval; * represents significant differences compared to the corresponding untreated control group (w/o TGFβ3) (*p* < 0.05).

Macroscopically assessed morphological changes in form of cell-mediated scaffold contractions were present in all seeded scaffolds (Figure 1a). The addition of TGFβ3 via both application forms increased the level of morphological alterations. This TGFβ3-driven effect was significant for scaffolds that were directly loaded with TGFβ3 at days 3 and 5 (*p* < 0.05).

#### 2.2.2. Cell Distribution and Cell Integration

**Figure 2:**
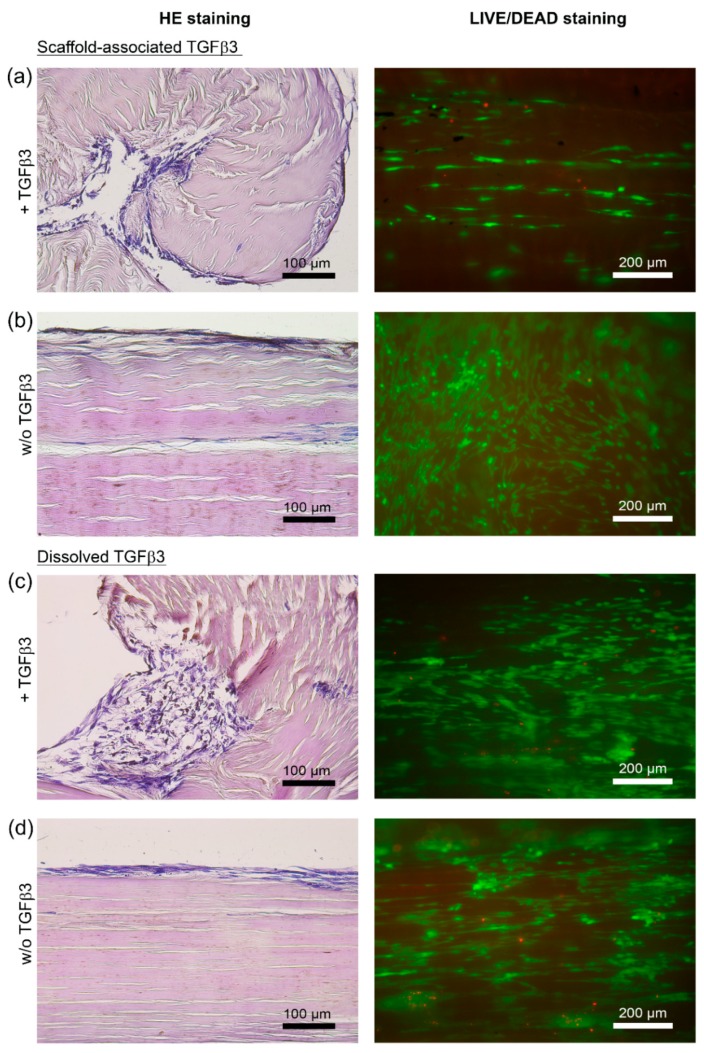
Microscopic appearance of MSC-seeded tendon scaffolds treated with TGFβ3 (+TGFβ3) and the respective internal control scaffolds (w/o TGFβ3). Representative images of hematoxylin- and eosin-stained paraffin sections of MSC-seeded tendon scaffolds (**a**–**d**, left) and of corresponding LIVE/DEAD^®^-stained MSC-seeded tendon scaffolds (**a**–**d**, right). The panel of LIVE/DEAD^®^-stained scaffolds shows vital cells in green and cells with defect cellular membranes in red. MSC-seeded tendon scaffolds were either directly loaded with TGFβ3 (scaffold-associated) (**a**), or TGFβ3 was applied as a standard cell culture medium supplement (dissolved) (**c**). Respective internal control scaffolds—(**b**) internal control for scaffolds directly loaded with TGFβ3; (**d**) internal control for scaffolds that received TGFβ3 as a standard cell culture medium supplement—were not treated with TGFβ3 (w/o TGFβ3). Note the obvious alterations of the scaffold morphology (left panel of hematoxylin- and eosin-stained sections), illustrating the increased cell-mediated scaffold contractions in the presence of TGFβ3 regardless of the route of application ((**a**) directly applied TGFβ3 and (**c**) TGFβ3 applied as a cell culture medium supplement). All images shown were taken after 5 days from tendon scaffolds that were seeded with MSC from the same donor horse.

The microscopic evaluation of hematoxylin- and eosin-stained longitudinal sections showed that all seeded scaffolds displayed a moderate cell distribution of roughly 3 score points, which corresponds to a cell distribution pattern of mainly focally clustered cells. The application of TGFβ3 did not alter the results regarding cell distribution (Figure 1b and Figure 2).

The cell integration into deeper scaffold layers was low in all evaluated scaffolds, with 10% or less integrated cells (represented by 2 score points). Seeded scaffolds treated with TGFβ3, and especially scaffolds directly loaded with TGFβ3, tended to be assigned lower score points (Figure 1c and Figure 2) compared to their respective internal controls (w/o TGFβ3).

#### 2.2.3. Cell Shape and Viability

**Figure 3:**
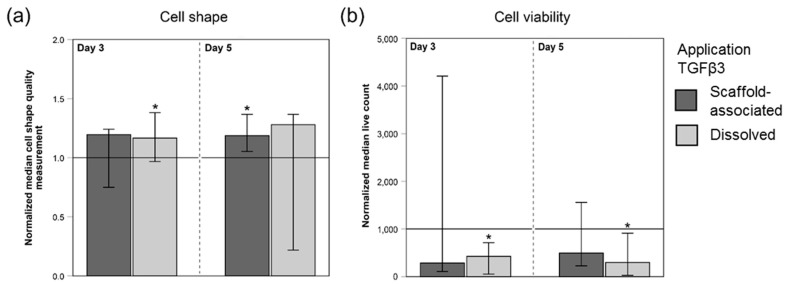
Quantitative image analysis results of LIVE/DEAD^®^-stained, MSC-seeded tendon scaffolds. Values of the cell shape measurements (**a**) and of the numbers of viable cells (**b**) were normalized to the corresponding internal controls (w/o TGFβ3; indicated by the horizontal line intersecting the *x*-axis at 1.0). Higher values of the cell shape measurement in (**a**) correspond to more elongated cells. MSC-seeded tendon scaffolds were either directly loaded with TGFβ3 (scaffold-associated) or TGFβ3 was supplemented via the cell culture medium (dissolved). The total number of scaffolds within each group was 42 (*n* = 42). Bars represent the normalized median values and error bars the 95% confidence interval; one extreme outlier value was removed (scaffold-bound TGFβ3 group in (**b**), day 3) before plotting the graph; * illustrates significant differences compared to the respective untreated control group (w/o TGFβ3) (*p* < 0.05).

Evaluation of cell shapes indicated that TGFβ3 led to more elongated cells at days 3 and 5 (Figure 3a). This difference in the cellular appearance between TGFβ3-treated MSC and internal control MSC (w/o TGFβ3) was significant at day 3 for MSC treated with dissolved TGFβ3 and at day 5 for MSC seeded on scaffolds directly loaded with TGFβ3 (*p* < 0.05).

The numbers of viable cells tended to be higher in internal control scaffolds (w/o TGFβ3) at days 3 and 5. This TGFβ3-induced slower MSC growth indicated by lower numbers of viable cells was significant in scaffolds treated with dissolved TGFβ3 at day 3 and 5 (*p* < 0.05) (Figure 3b).

#### 2.2.4. TGFβ3-Mediated Effects on the Gene Expression of ECM Molecules

**Figure 4:**
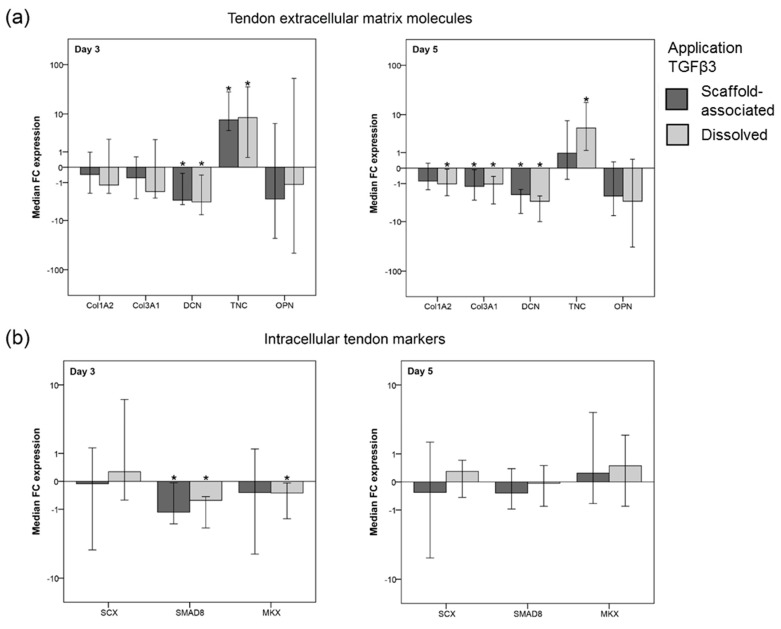
Gene expression levels of tendon extracellular matrix (ECM) molecules (**a**) and intracellular tendon markers (**b**) in tendon scaffold-seeded MSC. Data are given as “fold change” (FC) to the respective internal control (w/o TGFβ3), which is displayed as a horizontal line intersecting the *x*-axis at zero. Tendon scaffolds seeded with MSC were either directly loaded with TGFβ3 (scaffold-associated) or supplemented with TGFβ3 added to the cell culture medium (dissolved). Part (**a**) includes the gene expression of collagen 1A2 (Col1A2), collagen 3A1 (Col3A1), decorin (DCN), and tenascin c (TNC), as well as the expression of osteopontin (OPN), which is related to osteogenic differentiation. Part (**b**) shows the gene expression of scleraxis (SCX), smad8 (SMAD8), and mohawk (MKX). The total number of scaffolds within each group was 42 (*n* = 42). Bars represent the median fold changes values and error bars the 95% confidence interval; * illustrates significant differences compared to the untreated control (w/o TGFβ3) (*p* < 0.05).

Both scaffold-associated and dissolved TGFβ3 induced an upregulation of tenascin c expression (*p* < 0.05 at day 3 for both application forms and at day 5 for dissolved TGFβ3). Other ECM genes were downregulated in the presence of TGFβ3, which was significant for decorin at day 3 and 5 (*p* < 0.05 for both application forms) and at day 5 also for collagen 1A2 (*p* < 0.05 for dissolved TGFβ3) and collagen 3A1 (*p* < 0.05 for both application forms) (Figure 4a). Collagen 2A1 expression was not detected in any sample.

#### 2.2.5. TGFβ3-Mediated Effects on the Gene Expression of Intracellular Tendon Markers

The fold change expression of the intracellular tendon markers scleraxis, smad8 and mohawk showed an overall inhomogeneous pattern with only a few consistently observed TGFβ3 effects (Figure 4b). These included a moderate, albeit significant, downregulation of smad8 (*p* < 0.05 for both application forms) and mohawk (*p* < 0.05 for dissolved TGFβ3) at day 3 (Figure 4b).

## 3. Discussion

The current study presents a functionally engineered tendon ECM-based construct that was loaded with recombinant TGFβ3 as a possible future growth factor delivery system in tendon tissue engineering. Growth factors like TGFβ3 represent a crucial source of biomolecular cues in any tissue engineering approach [6]. Until now, TGFβ3-loaded ECM-derived scaffolds have been described for in vitro models mimicking cartilage tissue and being conducive to chondrogenic differentiation [36,37,38]. However, to the best of our knowledge, the current and one previously published study of our group are among the first to evaluate the application of TGFβ3 absorbed in a native tendon ECM-based construct [39]. TGFβ3 was successfully loaded onto scaffold surfaces. Furthermore, the present study shows that the bioactivity of scaffold-associated TGFβ3 with regard to its tenoinductive effect on scaffold-seeded MSC is preserved. Therefore, TGFβ3-loaded and MSC-seeded decellularized tendon scaffolds potentially represent a promising therapeutic tool for future applications in human tendon defects, justifying further characterization of the putative tissue-engineered product.

Direct approaches to immobilize growth factors like TGFβ3 to biomaterials include physical immobilization techniques, nonselective covalent immobilization procedures through functional residues as well as immobilization techniques that are based on the specific bioaffinity of the respective growth factors [9]. The present study used the physical immobilization technique of absorption over a period of 24 h to load TGFβ3 onto decellularized tendon scaffolds. This methodological approach is frequently used for diverse types of scaffolds due to its feasibility under mild circumstances at room temperature. However, it has to be taken into account that an inadequate storage of stable soluble growth factors within the scaffold material and a poorly controlled growth factor delivery are frequently reported issues [9,40]. By loading the growth factor directly onto the scaffold, a narrow niche is created, allowing for interactions of scaffold-associated TGFβ3 with both the tendon ECM components as well as the seeded MSC. Thereby, early conformational changes and unpredictable interactions induced by ingredients of the cell culture medium could be reduced and a close spatial relationship of TGFβ3 and MSC is facilitated. It is well-known that secreted latent TGFβ3 (complex of mature TGFβ3, latency-associated peptide, and latent TGFβ3 binding protein) represents not only an integral part of the ECM, but also a binding target for integrins. Therefore, it partly creates the interface between cellular fate determination and the regulating ECM composition [41,42].

While the current study focused on TGFβ3 bioactivity, further investigations should address the spatiofunctional relationship between both the tendon ECM and the applied TGFβ3 in vitro. In particular, future studies should analyze the structural relationship between the tendon scaffold and directly applied TGFβ3 to show, if scaffold-associated TGFβ3 forms defined bonds to molecules of the tendon ECM. Additionally, further evaluations should include release kinetics of scaffold-associated TGFβ3 and mechanisms by which TGFβ3 is released to further evaluate the decellularized tendon scaffolds as a potential TGFβ3 delivery system for functional tendon replacement in vivo. With regard to an in vivo application, additional investigations should also include preservation techniques that allow further storage for the presented tendon ECM-based construct.

In the current study, dissolved TGFβ3 was quantified using a commercial solid-phase sandwich ELISA kit, an established and sensitive method of analysis for nearly 20 years [43]. This showed that the percentage of scaffold-associated TGFβ3 ranged between 88–94%. However, the discrepancy in the amount of TGFβ3 used to determine scaffold loading efficiency (5 ng and 10 ng TGFβ3 per scaffold, chosen based on the dynamic range of the utilized ELISA kit) and to load the scaffolds for bioactivity assays (300 ng TGFβ3 per scaffold) represents a limitation of the current study. Future studies should include evaluations of a potential growth factor saturation or rather of a potential maximal TGFβ3 loading capacity of the prepared tendon scaffolds. Methodologically, the main limiting factor is the indirect determination of scaffold-associated TGFβ3 by measuring TGFβ after a 24 h incubation in the rinsing fluid of the loaded tendon scaffolds. Therefore, additional studies should not only specify the structural interplay of TGFβ3 and the tendon scaffold ECM, but also quantify scaffold-associated TGFβ3 directly including the normalization to the ECM dry matrix weight.

Demonstrating the preserved bioactivity, scaffold-associated and dissolved TGFβ3 had widely the same effects on scaffold-seeded MSC. TGFβ3 applied via both routes mediated an increase in the expression of the tendon ECM component tenascin c [32,44,45]. Although the expected TGFβ3-induced upregulation of collagen 1A2 failed to appear, we believe that the TGFβ3-mediated changes in the expression of tendon-related genes might represent a specific adjustment to the tendon microenvironment [39]. For example, the expression of the proteoglycan decorin was significantly decreased in the presence of TGFβ3. Against the background of decorin being expressed in MSC monolayer cultures and being even more highly expressed in MSC cultured on tendon scaffolds, TGFβ3 represents a potent regulator of the decorin expression in scaffold-seeded MSC and may enable an appropriately well-balanced decorin level [32,46,47].

It has to be taken into account that the relevance of smad8 for the tenogenic differentiation of scaffold-seeded MSC in the presence of TGFβ3 cannot be assessed conclusively in the current study because the amount of phosphorylated (active) smad8 was not analyzed. This would be of interest since active smad8 has been repeatedly reported to be a tenogenic factor, but until now there has been no clear proof of its activation upon TGFβ-superfamily member receptor binding and the tenogenic induction mediated by active smad8 [48,49,50]. Future studies should further evaluate the gene expression and activation of additional intracellular signaling molecules like SMAD2/3 to analyze the signaling pathway of TGFβ3 during tenogenic induction of MSC under different cell culture conditions.

Histologically, scaffold-associated as well as dissolved TGFβ3 significantly decreased cell proliferation, which is in accordance with the overall anti-proliferative effect of the three mammalian TGFβ isoforms [51]. Furthermore, TGFβ3-mediated histomorphological changes towards a tenocyte-like phenotype were evident for both scaffold-associated and dissolved TGFβ3. Finally, scaffold-associated TGFβ3, in particular, increased the MSC-mediated scaffold contraction. This could be part of a positive feedback mechanism, as the force generated by a contracting cell could shift integrin-bound latency associated peptide in ECM-stored latent TGFβ to unveil the hidden mature TGFβ and therefore allow TGFβ to bind to surface receptors of the same or neighboring cells [52]. Furthermore, a differentiation of the scaffold-seeded MSC into the myofibroblast lineage with contractile characteristics could not be excluded. A detailed interpretation of the TGFβ3-induced tenogenic effects on equine adipose tissue-derived MSC, which were seeded onto TGFβ3-loaded tendon scaffolds in vitro as well as cultured in a 2D monolayer with TGFβ3 supplementation, was recently published by our group [44].

Among clinically used native tendon-based scaffolds in human medicine, the implantation of xenogeneic constructs has great impact due to financial reasons and potential donor site morbidity [27]. Equine tendon tissue, as presented in this study, is among the most frequently researched xenogeneic tissues sources [53]. Additionally, in terms of translational aspects, the equine superficial digital flexor tendon represents the functional and clinical equivalent to the human Achilles tendon and thereby displays useful properties as model of human tendinopathies [54]. Due to the diversity of microarchitecture, type, and size of tendon lesions that is further influenced by age and mobility of the patients, there is a growing need for personalized and effective treatments in tendon injuries. The present study provides a first methodological prospect for the application of TGFβ3 absorbed in decellularized equine tendon tissue and could be the starting point for the development of functionalized scaffolds for future tendon tissue engineering approaches. This could provide a basis for future multiple growth factor delivery systems that could provide case-specific benefits allowing for personalized and effective treatment of human tendon pathologies [9,13,55,56].

## 4. Materials and Methods

### 4.1. Tendon Scaffolds

Tendon scaffolds were prepared from equine superficial digital flexor tendon specimens obtained at an abattoir [32,57]. Particular attention was given to aseptic working conditions throughout the whole scaffold preparation procedure, which included that freshly collected tendons were washed twice in ethanol. The decellularization protocol for full-thickness equine tendon samples comprised five repeated freeze-thaw cycles in liquid nitrogen, a 48 h incubation in hypotonic solution (distilled water), a 48 h incubation in 1M Tris buffer (Carl Roth GmbH & Co KG, Karlsruhe, Germany) containing 1% Triton X-100 (Carl Roth GmbH & Co KG, Karlsruhe, Germany) (pH: 7.6) as well as several washing steps (two consecutive 15 min washing steps in distilled water, a 24 h washing step in standard cell culture medium, a 24 h washing step in phosphate buffered saline (PBS)). The effectiveness of this protocol of decellularization has already been described (residual cell content of 1%, residual DNA content of 20%) [58]. Decellularized tendon samples (cut to the size of 10 mm × 10 mm) either maintained their natural thickness, or were customized to a thickness of 0.3 mm by using a cryostat (CM 3050 S; Leica Microscope CMS GmbH, Wetzlar, Germany). A thickness of 0.3 mm was chosen with regard to tendon tissue engineering approaches since the mechanical properties of 0.3 mm tendon slices remain nearly unchanged [35].

### 4.2. TGFβ3 Dilutions and Scaffold Loading

TGFβ3 (recombinant, human, CHO-expressed, carrier free; R&D Systems Inc., Minneapolis, MN, USA) was dissolved in PBS containing 1% bovine serum albumin (BSA) (herein referred to as RD (Reagent Diluent Concentrate 2 (1x); R&D Systems)). First, to evaluate possible TGFβ3 trapping by the surface of standard cell culture dishes, 5 ng or 10 ng TGFβ3 in 1 mL RD were incubated for 24 h (37 °C, humidified 5% CO_2_ atmosphere) in dishes with a surface frequently used for adherent cell culture (CELLSTAR^®^, cell culture multiwell plate, 24 wells, polystyrene, tissue culture surface treatment; Greiner Bio One International GmbH, Kremsmünster, Austria). Dishes with a surface inhibiting cellular attachment and protein absorption (Corning^®^ Costar^®^ Ultra-Low attachment multiwell plate, 24 wells, polystyrene; Corning Inc., Corning, NY, USA), reported to prevent an unspecific surface binding of TGFβ3 by providing a hydrophilic and uncharged surface [59], were used as reference and control. After the 24 h incubation, TGFβ3 was measured by ELISA (Human TGF-beta 3 DuoSet ELISA; DuoSet ELISA Ancillary Reagent Kit 2; R&D Systems Inc., Minneapolis, MN, USA).

Next, to assess the efficiency of scaffold loading with TGFβ3, the scaffolds were placed into ultra-low attachment 24-well plates (one scaffold per well), which were prepared with 0.1 mL RD per well, to allow easy placement of the tendon scaffolds and to prevent their drying-out. Subsequently, an amount of 5 ng or 10 ng TGFβ3 was dissolved in a volume of 30 µL RD and pipetted directly onto the scaffold surface in a meandering loop pattern. A precise application of TGFβ3 was ensured by careful drop by drop pipetting so that there was no leakage of the pipetted growth factor solution and the scaffold surface was homogeneously loaded with TGFβ3. Scaffolds were incubated for 24 h in the covered plate (37 °C, humidified 5% CO_2_ atmosphere) and then rinsed in 0.9 mL RD to dissolve unbound TGFβ3. The rinsing solutions were again measured by ELISA.

To evaluate the bioactivity of scaffold-bound TGFβ3, customized tendon scaffolds (10 mm × 10 mm × 0.3 mm) were loaded with 300 ng TGFβ3 dissolved in 30 µL RD as otherwise described above. Corresponding control scaffolds (w/o TGFβ3) received 30 µL RD without any TGFβ3 supplementation. Immediately after the 24 h incubation, TGFβ3-loaded scaffolds were then seeded with MSC as described below and incubated in standard culture medium without further TGFβ3 supplementation. To compare the bioactivity of scaffold-associated TGFβ3 with dissolved TGFβ3, untreated decellularized tendon scaffolds were seeded with MSC accordingly and the same amount of TGFβ3 (300 ng dissolved in 30 µL RD) was then added to the total volume of 1 mL standard cell culture medium for each scaffold. The standard cell culture medium of the corresponding control scaffolds (w/o TGFβ3) was supplemented with 30 µL RD only. Seeded scaffolds were subjected to analyses of tenogenic TGFβ3 effects as described below.

### 4.3. Quantification of TGFβ3 by ELISA

Samples were analyzed by a solid phase sandwich ELISA kit (Human TGF-beta 3 DuoSet ELISA; DuoSet ELISA Ancillary Reagent Kit 2; R&D Systems Inc., Minneapolis, MN, USA) according to the manufacturer’s instructions. All optical density values of samples were obtained using a Synergy H1 Hybrid Multi-Mode Reader and the Gen5 2.0 software (BioTek Instruments Inc., Winooski, VT, USA). The percentage rates of TGFβ3 trapping by the cell culture dishes or TGFβ3 association to the scaffolds, respectively, were calculated based on the positive control values obtained after incubation of TGFβ3 dilutions in the ultra-low attachment dishes, which was set to 100%.

### 4.4. MSC Culture and Scaffold Seeding

Equine adipose tissue was collected from donor horses sacrificed for reasons unrelated to the present study. Therefore, in accordance with national guidelines and the local ethics committee (Landesdirektion 455 Leipzig, Germany), no license was required for sample collection. Equine MSC recovered by collagenase I digestion (0.8 mg/mL; Thermo Fisher Scientific/Life Technologies GmbH™, Waltham, MA, USA) were cultured until passage 3 in standard cell culture medium (Dulbecco’s modified Eagle medium 1 g/L glucose (Gibco^®^ by Life Technologies GmbH™, Waltham, MA, USA) supplemented with 10% fetal bovine serum (FBS; Gibco^®^ by Life Technologies GmbH™, Waltham, MA, USA), 1% penicillin-streptomycin (Sigma Aldrich, St. Louis, MO, USA) and 0.1% gentamycin (Carl Roth GmbH & Co KG, Karlsruhe, Germany)) (37 °C, humidified 5% CO_2_ atmosphere).

When reaching 80–90% confluence of the cell monolayer, MSC were subjected to a 24 h cell synchronization using standard cell culture medium supplemented with 1% FBS. Finally, the low-level FBS concentration was replaced, cells were again cultivated for 24 h in standard cell culture medium (containing 10% FBS) and were then detached enzymatically by trypsinization. MSC were then seeded onto the tendon scaffold surfaces (0.3 × 10^6^ MSC / 30 µL/1 cm^2^). They were allowed to attach for 6 h before the volume of 1 mL standard cell culture medium, with or without TGFβ3 supplementation according to the experimental groups described above, was added. Subsequently, all samples were incubated for 3 and 5 days (37 °C, humidified 5% CO_2_ atmosphere) and were finally subjected to the evaluation of TGFβ3-induced tenogenic effects on scaffold-seeded MSC to assess the bioactivity of the applied TGFβ3.

### 4.5. Macroscopic Assessment of the Scaffold Morphology

Morphological alterations due to cell-mediated scaffold contraction were evaluated macroscopically 3 and 5 days after scaffold seeding by two independent observers blinded to the experimental group. The applied semi-quantitative rating system included the score points 1 to 4. In the absence of morphological changes, scaffolds were assigned 1 score point. The number of the rolled-up scaffold edges determined the score points 2 (1 out of 4 edges are rolled up), 3 (2 or 3 edges out of 4 are rolled up), and 4 (all edges are rolled up).

### 4.6. Histology

For the assessment of the cell distribution and the cell integration into the scaffold, hematoxylin- and eosin-stained sections (paraffin-embedded; three longitudinal 5 µm sections from the central part of each sample) were evaluated microscopically (10× objective; Leica DMi1, Leica MC 170HD, Leica Microscope CMS GmbH, Wetzlar, Germany) by two independent observers blinded to the sample group. Both the semi-quantitative rating systems for the evaluation of the cell distribution (score point 1: no seeded cells; score point 2: isolated seeded cells; score point 3: focal cell clusters of seeded cells; score point 4: continuous and even cell layer of seeded cells), as well as of the cell integration (score point 1: no cell integration; score point 2: cell integration of < 10%; score point 3: cell integration of 10–50%; score point 4: cell integration of > 50%) included the score points 1 to 4.

Additionally, the parameters cell viability and cell shape quality of scaffold-seeded MSC were assessed via LIVE/DEAD^®^ staining of MSC-seeded scaffolds at day 3 and 5 [LIVE/DEAD^®^ Viability / Cytotoxicity Kit, for mammalian cells; Thermo Fisher Scientific, Waltham, MA, USA; calcein AM 4 mM in anhydrous DMSO, ethidium homodimer I 2 mM in DMSO/H_2_O 1:4 (*v*/*v*)]. Three regions of each stained scaffold were randomly chosen and digitally imaged at 4× objective (Keyence BZ 9000E, BZ II Analyzer 2.2 Software; Keyence Corporation, Osaka, Japan). To detect and quantify MSC in a consistent manner, a combined image processing chain was applied. The analysis of the cell shape was performed by an additional measure that favors elongated cells and penalizes round cells. All calculations were performed with Wolfram Mathematica version 11.1 (Wolfram Research, Inc., 2017, Champaign, IL, USA) [39].

The preprocessing consisted of a difference-of-Gaussian (DoG) filter to enhance cell features and to reduce the global illumination inconsistencies, and a total variation (TV) to remove noise without smoothing significant cell edges. Global thresholding was employed to separate cells from the background, and a connected component analysis identified separate cells in the image. For each cell, several morphological measurements were computed, i.e., the area, the cell orientation, the bounded disk coverage, and the elongation of the cell. To obtain only valid cells for the analysis, the area measure was employed to remove image components that are too small. Furthermore, as the processing chain enhances features in very dark regions, the mean pixel value of the component was used to remove invalid elements. For all remaining cells, the above-mentioned morphological measures were calculated. To find correct values for the filter parameters, the first author adjusted the image processing steps for a subset of ten representative images. The mean values of the found parameters were then utilized for all images.

### 4.7. Real-Time PCR

Gene expression analysis was performed by using real-time PCR. Included genes encode for tendon ECM molecules (collagen 1A2, collagen 3A1, decorin, tenascin c) as well as for intracellular tendon markers (scleraxis, smad8, mohawk). Additionally, the gene expression of collagen 2A1 and osteopontin was analyzed to evaluate potential chondrogenic and osteogenic induction of scaffold-seeded MSC. GAPDH and beta actin served as housekeeping genes. Table 2 shows the primer sequences.

Frozen tendon samples were homogenized with the Tissue Lyser II (Qiagen, Hilden, Germany) and subsequently treated with proteinase k (Qiagen, Hilden, Germany) at 55 °C. Afterwards, total RNA was isolated (RNeasy^®^ Mini Kit with On Column DNase digestion; Qiagen, Hilden, Germany), RNA was quantified using the NanoDrop2000 Spectrophotometer, and 1.5 µg of RNA was converted to first strand cDNA with Reverse Transcriptase RevertAid H Minus (Thermo Fisher Scientific/Life Technologies GmbH™, Waltham, MA, USA). cDNA was mixed with primers and iQ™ SYBR Green Supermix (Bio-Rad Laboratories, Hercules, CA, USA), and the relative quantification was carried out by an Applied Biosystems™ 7500 Real-Time PCR System Thermo Fisher Scientific/Life Technologies GmbH™, Waltham, MA, USA). Relative gene expression ratios were calculated using the Pfaffl method and normalized to those of the corresponding controls (scaffolds w/o TGFβ3 application) [60]. Obtained data are given as fold change increase ((FC_i_ = (ratio_treated_/ratio_control_) − 1) or decrease (FC_d_ = 1/(ratio_treated_/ratio_control_) − 1).

### 4.8. Statistical Analysis

Using SPSS^®^ Statistics 23.0 software (IBM Deutschland GmbH, Ehningen, Germany), Friedman tests and Wilcoxon signed rank tests were performed to examine differences between the experimental groups since the collected data were tested to be not normally distributed. Outlier tests to categorically exclude such values were not performed in order to fully represent the biological variability. Therefore, all collected and technically sound data were included in the statistical tests and shown in the figures. As an exception to this, one single value of counted viable cells (scaffold-associated TGFβ3 group on day 3) in Figure 3b was removed from the graph to improve its clarity (Figure 3b). Non-normalized data are shown in the Supplementary Files 1–7 as boxplots depicting the outlier values. For the final statistical analysis, median values of sample technical replicates and median values of the two independent observers were calculated. The level of significance was set to *p* = 0.05.

## Figures and Tables

**Table 1:** Quantification of TGFβ3 by ELISA. Optical densities (OD) of the TGFβ3 control solutions after 24 h incubation in ultra-low attachment or standard dishes and OD of the rinsing fluids obtained 24 h after scaffold loading. The percentages of dissolved and surface-trapped/scaffold-associated TGFβ3 were calculated based on the control measurements after incubation in ultra-low attachment dishes.

	Control TGFβ3 Solution		Rinsing Fluid after Scaffold Loading	
	Ultra-Low Attachment Dish (*n* = 7)	Standard Dish (*n* = 2)	Full-Thickness Scaffold (*n* = 2)	0.3 mm Scaffold (*n* = 3)
**5 ng TGFβ3:**				
**OD (mean ± SD)**	1.325 ± 0.322	0.426 ± 0.066	0.08 ± 0.005	0.139 ± 0.022
**Dissolved TGFβ3 (%)**	100 ± 24	32 ± 5	6 ± 0.4	10 ± 2
**Scaffold-Associated TGFβ3 (%)**		68 ± 5	94 ± 0.4	90 ± 2
**10 ng TGFβ3:**				
**OD (mean ± SD)**	2.391 ± 0.454	1.416 ± 0.000	0.194 ± 0.011	0.292 ± 0.050
**Dissolved TGFβ3 (%)**	100 ± 19	59 ± 0	8 ± 0.5	12 ± 2
**Scaffold-Associated TGFβ3 (%)**		41 ± 0	92 ± 0.5	88 ± 2

**Table 2:** Primer sequences for quantitative real-time PCR.

Equine Gene	Primer Pair Sequences	Accession Number	PCR Product in bp
Beta-actin	For: ATCCACGAAACTACCTTCAAC Rev: CGCAATGATCTTGATCTTCATC	NM_001081838.1	174
GAPDH	For: TGGAGAAAGCTGCCAAATACG Rev: GGCCTTTCTCCTTCTCTTGC	NM_001163856.1	309
Collagen 1A2	For: CAACCGGAGATAGAGGACCA Rev: CAGGTCCTTGGAAACCTTGA]	XM_001492939.3	243]
Collagen 2A1	For: ATTGTAGGACCCAAAGGACC Rev: CAGCAAAGTTTCCACCAAGG	NM_001081764.1	199
Collagen 3A1	For: AGGGGACCTGGTTACTGCTT Rev: TCTCTGGGTTGGGACAGTCT	XM_001917620.3	216
Scleraxis	For: TACCTGGGTTTTCTTCTGGTCACT Rev: TATCAAAGACACAAGATGCCAGC	NM_001105150.1	51
Osteopontin	For: TGAAGACCAGTATCCTGATGC Rev: GCTGACTTGTTTCCTGACTG	XM_001496152.3	158
Decorin	For: ACCCACTGAAGAGCTCAGGA Rev: GCCATTGTCAACAGCAGAGA	NM_001081925.2	239
Tenascin c	For: TCACATCCAGGTGCTTATTCC Rev: CTAGAGTGTCTCACTATCAGG	XM_001916622.3	163
Mohawk	For: AAGATACTCTTGGCGCTCGG Rev: ACACTAAGCCGCTCAGCA	XM_014737017.1	170
Smad8	For: AGCCTCCGTGCTCTGCATT Rev: CCCAACTCGGTTGTTTAGTTCAT	AB106117.1	200

## Supplementary Materials

Supplementary materials can be found at https://www.mdpi.com/1422-0067/20/21/5474/s1.

## Abbreviations

TGFβ3	Transforming growth factor beta 3
ECM	Extracellular matrix
MSC	Mesenchymal stromal cells
PBS	Phosphate buffered saline
BSA	Bovine serum albumin
FBS	Fetal bovine serum
