# Supplementary material for: Transforming Growth Factor Beta 3-Loaded Decellularized Equine Tendon Matrix for Orthopedic Tissue Engineering
Source: Int J Mol Sci. 2019 Nov 3;20(21):5474. doi: 10.3390/ijms20215474 (PMC6862173; doi:10.3390/ijms20215474)
Supplement: Supplementary file 1 [file ijms-20-05474-s001.pdf]

Supplementary Files

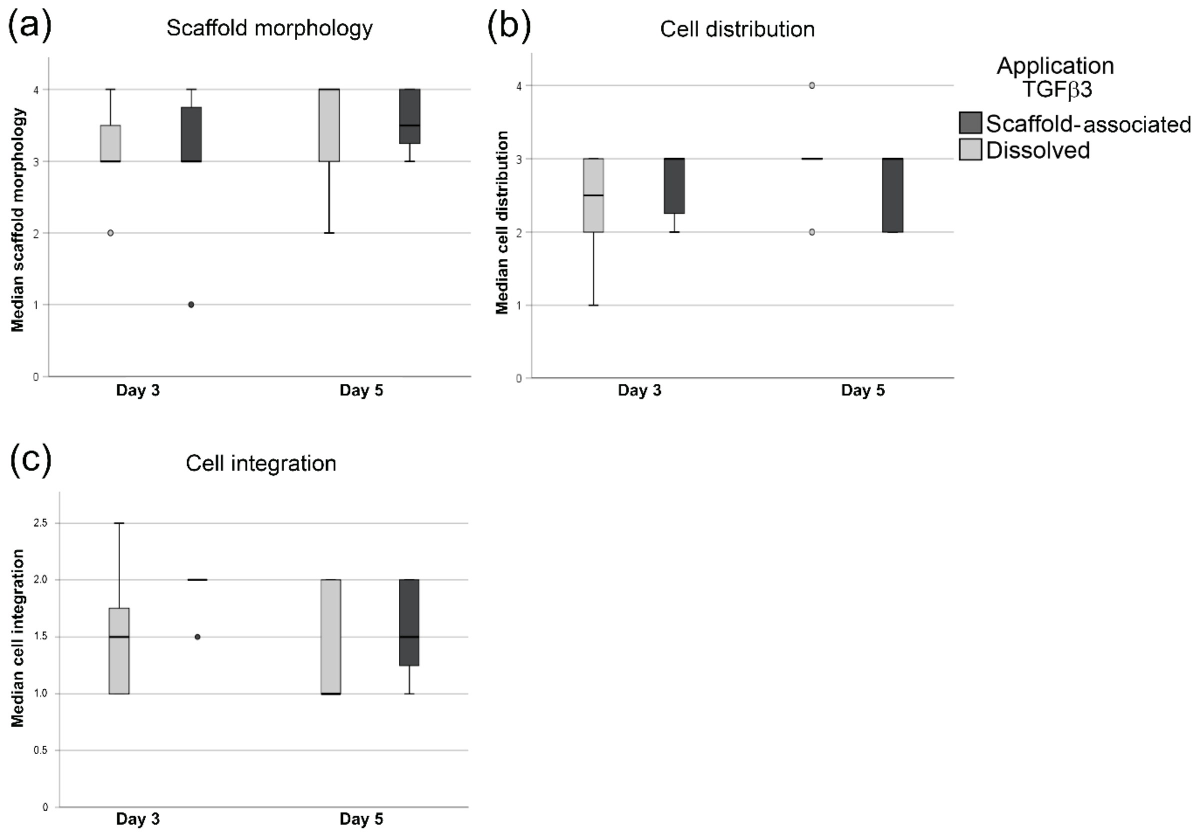

Supplementary File 1. Median score points given to evaluate the macroscopic tendon scaffold morphology **(a)**, cell distribution **(b)**, and cell integration **(c)** in the presence of TGFβ3 on day 3 and on day 5. TGFβ3 was either directly loaded on tendon scaffold specimens (scaffold-associated TGFβ3) or applied as a cell culture medium supplement (dissolved TGFβ3). Evaluation of the macroscopic scaffold morphology **(a)**, cell distribution **(b)**, and cell integration **(c)**, was performed by two independent observers blinded to the experimental group. Data are depicted as boxplots, circles represent outlier values.

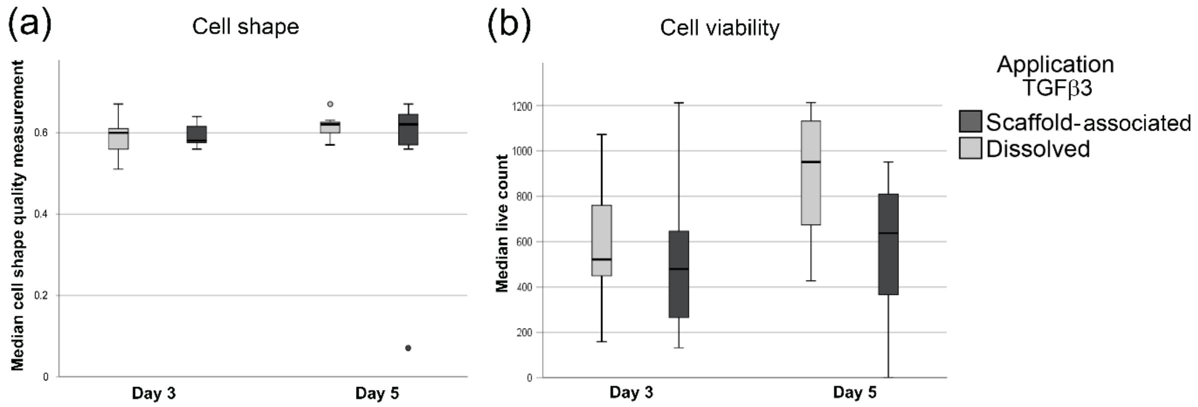

Supplentary File 2. Median data values of the cell shape quality measurement **(a)** and number of viable cells **(b)** to evaluate the cell shape the cell viability of MSC seeded on tendon scaffolds in the presence of TGFβ3 on day 3 and on day 5. TGFβ3 was either directly loaded on tendon scaffold specimens (scaffold-associated TGFβ3) or applied as a cell culture medium supplement (dissolved TGFβ3). LIVE/DEAD® staining of MSC-seeded scaffolds was performed and three randomly chosen regions of each stained scaffold sample were digitally imaged at 4xmagnification. Cell detection and quantification was ensured by different filters. To quantify the cell shape, a linear combination of two parameters was determined and applied for each detected scaffold-seeded cell. The higher the calculated value (cell shape quality measurement), the more elongated was the cell shape of the scaffold-seeded cell. Data are depicted as boxplots, circles represent outlier values.

Tendon extracellular matrix molecules

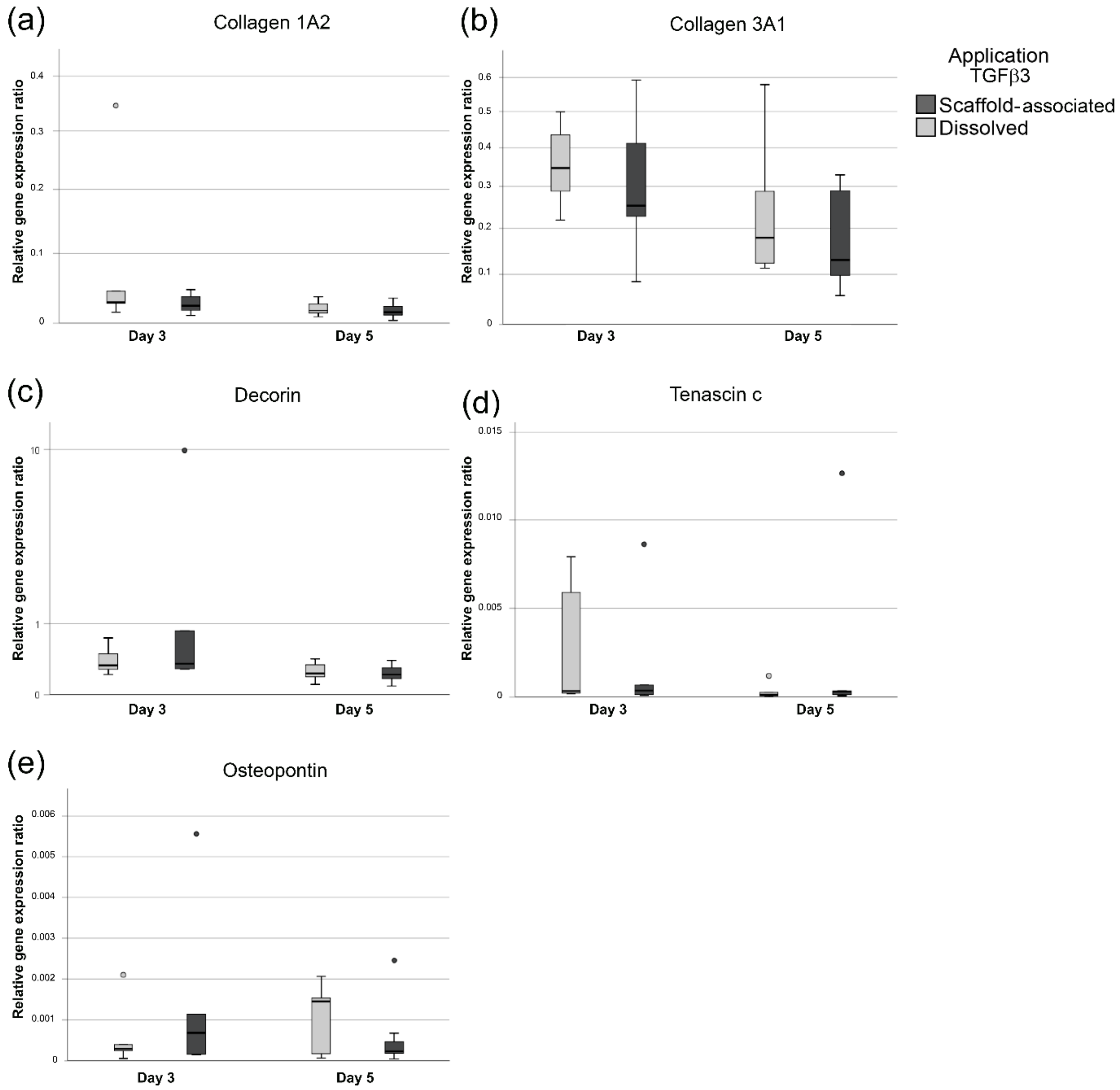

Supplementary File 3. Relative gene expression ratio of the tendon extracellular matrix molecules **(a)** collagen 1A2 (Col1A2), **(b)** collagen 3A1 (Col3A1), **(c)** decorin (DCN), **(d)** tenascin c (TNC), and **(e)** osteopontin (OPN) of MSC seeded on tendon scaffolds in the presence of TGFβ3 on day 3 and on day 5. TGFβ3 was either directly loaded on tendon scaffold specimens (scaffold-associated TGFβ3) or applied as a cell culture medium supplement (dissolved TGFβ3). The logarithm of the relative gene expression ratio is depicted as boxplot, circles represent outlier values.

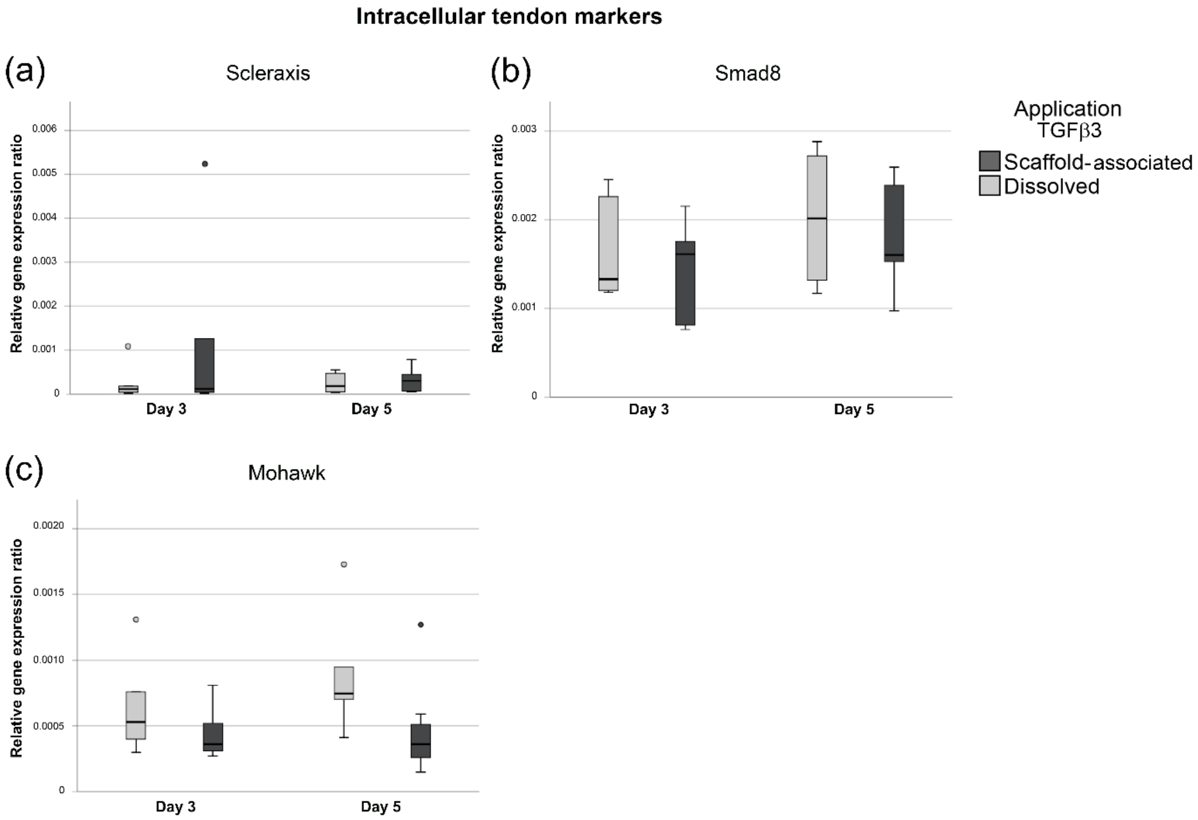

Supplementary File 4. Relative gene expression ratio of the intracellular tendon markers **(a)** scleraxis (SCX), **(b)** smad8 (SMAD8), and **(c)** mohawk (MKX) of MSC seeded on tendon scaffolds in the presence of TGFβ3 on day 3 and on day 5. TGFβ3 was either directly loaded on tendon scaffold specimens (scaffold-associated TGFβ3) or applied as a cell culture medium supplement (dissolved TGFβ3). The logarithm of the relative gene expression ratio is depicted as boxplot, circles represent outlier values.
